# A consolidation algorithm for genomes fractionated after higher order polyploidization

**DOI:** 10.1186/1471-2105-13-S19-S8

**Published:** 2012-12-19

**Authors:** Katharina Jahn, Chunfang Zheng, Jakub Kováč, David Sankoff

**Affiliations:** 1 Department of Mathematics and Statistics, University of Ottawa, 585 King Edward Avenue, Ottawa, Canada K1N 6N5

## Abstract

**Background:**

It has recently been shown that fractionation, the random loss of excess gene copies after a whole genome duplication event, is a major cause of gene order disruption. When estimating evolutionary distances between genomes based on chromosomal rearrangement, fractionation inevitably leads to significant overestimation of classic rearrangement distances. This bias can be largely avoided when genomes are preprocessed by "consolidation", a procedure that identifies and accounts for regions of fractionation.

**Results:**

In this paper, we present a new consolidation algorithm that extends and improves previous work in several directions. We extend the notion of the fractionation region to use information provided by regions where this process is still ongoing. The new algorithm can optionally work with this new definition of fractionation region and is able to process not only tetraploids but also genomes that have undergone hexaploidization and polyploidization events of higher order. Finally, this algorithm reduces the asymptotic time complexity of consolidation from quadratic to linear dependence on the genome size. The new algorithm is applied both to plant genomes and to simulated data to study the effect of fractionation in ancient hexaploids.

## Background

Polyploidy, a genetic property whereby some *k *> 1 copies of each chromosome co-occur in the haploid genome, is widespread in flowering plants, and usually characterizes a distinct species, non-interbreeding with the diploid (*k *= 1) plant containing the same chromosomes, if this exists, or with plants with any other *k' *≠ *k*. Tetraploids (*k *= 2) are particularly common, but octoploids (*k *= 4) and hexadecaploids (*k *= 8) also occur, as do hexaploids (*k *= 3), decaploids (*k *= 5), dodecaploids (*k *= 6) and other multiplicities^*a *^. Polyploidization is considered a mutation, and is not part of the normal life cycle of plants, or of the natural history of a population, though *sympatric *populations of different ploidies may occasionally originate in the same geographical area, and share the same territory.

Over evolutionary time, polyploids may undergo rediploidization. The *k homeologous*, or originally identical, chromosomes, diverge in DNA sequence, in gene content and gene order, and gross chromosomal structure, through various processes such as chromosome fission or fusion, inversion of chromosomal fragments, translocations of chromosome arms or other fragments from one chromosome to another. A focus in this paper is *fractionation *[1], the eventual loss of most duplicate genes after polyploidization. This may cause more gene order disruption than classical chromosomal rearrangements such as inversion or reciprocal translocation. Although polyploidization, mainly tetraploidization, is known to have occurred in ferns and other vascular plants, in yeasts and other fungi, in goldfish and salmon, in *Paramecium *and other protists, and even in a mammal, polyploidization followed by fractionation are particularly prevalent in the history of the flowering plants [2], where the alternation of the two processes also necessitates the excision of excess non-coding DNA [3,4], a major difference between these organisms and some other evolutionary domains, such as the mammals.

The evolutionary disruption of gene order caused by fractionation in a polyploid is a result of the partly random choice of which of the *k *copies is deleted, i.e., which of *k *homeologous chromosomes retain their copy of the gene. This process was hypothesized by Wolfe and Shields, who discovered the ancient tetraploidization of *Saccharomyces cerevisiae *[5], and further studied through the comparison of the *S. cerevisiae *gene order with that of related diploid yeasts [6,7].

When the polyploid history of a diploid genome is discovered through the detection of such interleaving patterns plus the retention of the occasional remaining duplicate, triplicate, etc., gene pair in formerly homeologous regions of the genome (since fragmented and rearranged), the diploid is often called an *paleopolyploid *or simply an ancient polyploid.

**Figure 1:**
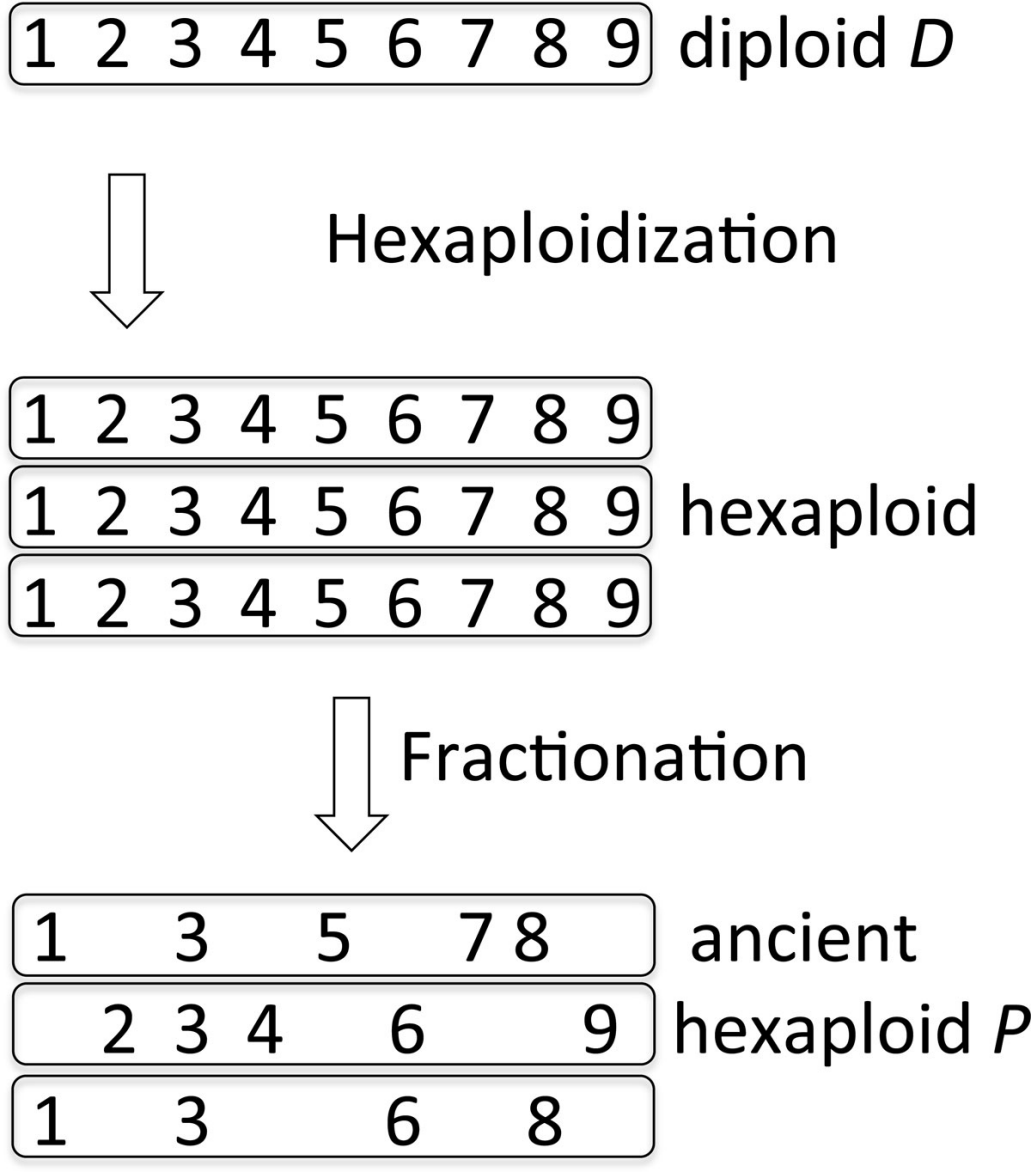
Fractionation leading to different adjacencies in diploid and ancient hexaploid

Fractionation deletes redundant genes from the *k *homeologs in an arbitrary way, as long as at least one copy of each *k*−tuple is retained, as in Figure 1. Methods for inferring the rearrangement distances between the paleopolyploid *P *(referred to as an "ancient polyploid" despite being a present-day genome, long since re-diploidized) and an unduplicated sister genome *D *automatically infer that there are rearrangement breakpoints where adjacency no longer exists between single-copy survivors, since the latter are on different chromosomes. This inflates the apparent number of reciprocal translocations, greatly exaggerating the overall amount of chromosomal rearrangement that has taken place in the two sister genomes. A goal of our work is to be able to computationally detect, characterize and correct for this impediment to the study of evolution.

Our method is based on the identification and isolation of "fractionation intervals", regions in both the ancient polyploid and its sister diploid that may have been rearranged internally, but have (so far) been unaffected by rearrangements exchanging genes from within the interval and genes external to the interval. A second objective of our work is to inventory these regions across the two genomes so that they can be studied quantitatively. The statistical properties of the intervals bear on current topics of interest in plant evolutionary genomics, whether duplicated genes are silenced or deleted one by one or through the deletion of longer stretches of DNA, whether a fractionation region tends to lose genes largely from one or two of the *k *homeologous chromosomal segments or equally from all of them, and on the question of subgenome dominance, i.e., whether any such bias towards one or some of the homeologs persists from the original polyploidization event and is unaffected by chromosome shuffling.

In previous work on ancient tetraploidy [8], we proposed a procedure for "consolidation" of two fractionated regions in the ancient tetraploid that correspond to a single region in a sister diploid. These are then represented by a single "virtual" gene, identical in the diploid and in two copies in the ancient tetraploid, removing the cause of excess rearrangement in the comparison of the two genomes. In the present work, we devise a new consolidation algorithm that can analyze polyploidy of any order (*k *= 2, 3, …), not just tetraploidy. Its running time is asymptotically linear in the genome size, improving on the previous quadratic time method. As the default option in its implementation, it analyzes fractionation regions that are only bounded by rearrangement breakpoints, but it can also consider the formulation in the previous work, where fractionation regions were bounded either by breakpoints or duplicated genes.

One of the most significant evolutionary events in the Tree of Life was a hexaploidization that occurred in the eudicot lineage of flowering plants, some 150 million years ago [9]. Soon after this event, there was a massive radiation of plant species, giving rise today to some 200,000 different species, which provide humans with the large majority of the familiar fruit and vegetables we consume.

This event, along with other hexaploidizations (e.g., tomato), octoploidizations and decaploidizations (e.g., strawberry), motivate our extensions and improvements of the current consolidation method.

Then to illustrate our method, we will simulate the rearrangement and fractionation of one ancient polyploid genome, that of the grapevine, following the hexaploidization event. Unfortunately, all of the published flowering plant genomes are descendants of this event or descendants of a more complicated series of polyploidizations in the monocot clade, so that there is no extant genome unaffected by polyploidization to compare grapevine with. We do, however, know the number of chromosomes in the pre-hexaploid ancestor, and can estimate the number of genes it contained [10]. We can then use these parameters in our simulations to investigate the relationship between fractionation and real versus apparent genome rearrangement. In addition, we can compare our algorithm versus the previous method in the comparison of grapevine with poplar, an ancient tetraploid.

## Methods

### Problem definition

For the original consolidation algorithm a fractionated region was defined as an interval *I*_1 _on a diploid reference genome *D *whose genes occur distributed over two intervals, *I*_2 _and *I*_3 _(the latter possibly empty), on an ancient tetraploid genome *P *such that *I*_1 _= *I*_2 _∪ *I*_3_, *I*_2 _∩ *I*_3 _= ∅ and all genes of *I*_1 _occur as single-copy genes in *P*. We extend this definition of a fractionation region in two directions: first we drop the requirement that *I*_2 _and *I*_3 _may not intersect. In doing so, it is possible to detect regions in which fractionation is still ongoing, as redundant gene copies may still be present in duplicated regions. Note that "intersection" refers to the gene sets *I*_2 _and *I*_3_, not physical overlaps of the index intervals that define the locations of *I*_2 _and *I*_3 _on *P*. (For obvious reason, the latter type of interval intersection is not allowed in context of fractionation.) Second we let genome *P *be of any type of ploidy *k *> 1 and therefore allow the genes from *I*_1 _to occur distributed over up to *k *different, non-physically overlapping intervals *I*_2_, ... *I*_*k*+1 _on *P*, as long as *I*_2 _∪ ... ∪ *I*_*k*+1 _= *I*_1 _holds, and none of the *I*_2_, ... *I*_*k*+1 _contains more than one copy of any gene from *I*_1_. The last condition reflects our goal to identify genomic regions that were shaped by fractionation. Any *k *+ 1-tuple (*I*_1_, *I*_2_, ... *I*_*k*+1_) that fulfills the above conditions is called a *virtual *gene.

The formal definition of the generalized fractionation problem is as follows:

Input: Triple (*L, D, P*), where

• *L *is a set of genes,

• *D *is a diploid genome with gene set *L*, i.e. *L *is partitioned among a number chromosomes, and the genes on the chromosomes are ordered.

• *P *is an "ancient" 2*k*-ploid genome with gene set *L*, i.e. each gene of *L *occurs in any copy number between 1 and *k*; the genes are partitioned and ordered on a number of chromosomes

Output: Triple (*L', D', P'*), where

• *D' *is a diploid over the gene set *L'*,

• *P' *is a "pure" 2*k*-ploid of *L' *(all genes occur exactly *k*-times),

• *L' *is a set of virtual genes whose intervals *I*_1 _define pairwise disjoint gene sets whose union is *L*.

### Preliminaries

Before we start with the description of the actual algorithm, we shortly revise the concept of *range minimum *and *range maximum *queries. Given two index positions *i *≤ *j *on a sequence *S *(like in our context a sequence of genes), a range minimum query returns the smallest element found in the interval on *S *that is bounded by the two positions. Likewise, a range maximum query returns the biggest number in this interval:

• *rMax*[*i*, *j*] = *max*{*S*[*i*], *S*[*i *+1], ..., S[*j*]},

• *rMin*[*i, j*] = *min*{*S*[*i*], *S*[*i *+1], ..., *S*[*j*]}.

Both types of queries can be answered in constant time after a preprocessing of *S *that takes time linear in its length [11,12].

For our algorithm we need the answers to the following range maximum and range minimum queries: For every index position *occ *that refers to an occurrence of a gene *g *and the up to *k *occurrences *occ' *of the next bigger gene *g' *occurring on the same chromosome (if such *occ' *exist), we need the range maximum and range minimum of the intervals [*occ, occ'*] (or [*occ' *, *occ*], if *occ' *<*occ*). With *k *being a constant, these values can be precomputed in linear time with respect to the size of *L*.

For the special case that every gene has at most one occurrence on each chromosome these above queries are uniquely defined for each chromosome position. In this case we refer to the above values as "range maximum of *occ*" and "range minimum of *occ*".

### Basic consolidation algorithm

We assume that the chromosomes of the diploid *D *are ordered and that the genes in *L *are named based on the order of their occurrence on *D*.

To simplify the initial description of the algorithm, we assume that no two copies of a gene are located on the same chromosome in *P*. The technical details that need to be added to the algorithm when this is not the case are discussed further below in a separate section.

The outline of the algorithm is the following: we iterate through all genes *g *in the order of their occurrence in *D *and add their occurrences on *P *one after the other to the list of already processed gene occurrences. As soon as we have added the last occurrence of a gene *g*, we test which virtual genes (*I*_1_, ..., *I*_*k*+1_) we can find that have the recently processed gene as biggest element. To do that we start from the occurrence most recently added to the list and then iterate backwards through the older list elements. The gene occurrences on *P *encountered in this process are distributed to the (initially empty) *I*_2_, ... *I*_*k*+1 _such that gene occurrences located on the same chromosome are always added to the same interval.

This process stops once all intervals *I*_2_, ... *I*_*k*+1 _have at least one element and we get to an occurrence that is located on the (*k *+ 1)-st different chromosome and there is no interval left to place it. This means that the gene occurrences accumulated in the backward search are distributed over too many chromosomes to form a virtual gene in a 2*k*-ploid genome. We also stop if we get to an occurrence whose range maximum value is bigger than the gene from which we started our backward search. This gene occurrence and the occurrence of the next bigger gene on the same chromosome cannot be together in the same interval *I_j _*unless the biggest gene located in between is also included. But this gene is bigger than the biggest gene in the current list, the gene from which we started the current backward search. The two occurrences may still be grouped together in a virtual gene but if so, this will only be determined in a backward search starting from a bigger gene.

The smallest gene in a virtual gene is detected in the backward search if the following holds: (*i*) We have reached the last occurrence of a gene, as a virtual gene needs to comprise all occurrences of any contained gene. (*ii*) During the backward search we encountered no gene occurrence with range minimum smaller than the gene represented by our current position in the backward search. Otherwise we need to continue the backward search at least until the gene occurrence representing this range minimum value is reached. If (*i*) and (*ii*) hold, each *I_j _*in (*I*_2_, ..., *I*_*k*+1_) is either empty (at most *k *− 1 of them) or the genes contained fall in the range between the gene where the backward search was started and the gene at the current position of the list. Also all occurrences of genes on *P *in this range are part of one of the intervals (*I*_2_, ..., *I*_*k*+1_). At this point, then, we have identified a virtual gene. Note that at least one virtual gene can be found for every start gene of the backward search, namely, the virtual gene that consists only of the occurrences of the starting gene itself. We maintain a list where we store for every backward search only the longest virtual gene. After all genes and their occurrences have been processed, we do a backward search over this list, adding only those intervals to *L' *that are not subintervals of the previously added interval. By construction, the virtual intervals form a partition of *L *. The algorithm as stated avoids multiple intervals on the same chromosome, although with little complication this can be removed.

### Backward search

The above algorithm is not yet linear in the genome size, as each backward search may process the whole list of already processed gene occurrences. In the following we show how this search can be done in constant time. Table 1 outlines the procedure.

To improve the backward search, we distinguish between terminal and non-terminal units in the list of processed gene occurrences. Terminal units are those gene occurrences that have been most recently added to a *I*_2_, ... *I*_*k*+1_. Their number is obviously bounded by *k*. A non-terminal unit can either be a single non-terminal gene occurrence or a consecutive stretch of non-terminal elements of the list that is represented by a single unit. Every gene occurrence is initially a terminal unit when added to the list and, unless it starts a new interval, some other terminal gene must lose this status. To update this element in constant time, we maintain a link from each terminal unit to the previous one. Following at most *k *− 1 links, we find the obsolete terminal unit and do the updates necessary to convert it into a non-terminal unit: in case the range minimum of the gene occurrence defined by the new non-terminal unit equals the gene represented by this occurrence, we set the value of *startPos *of this gene. If the range minimum is smaller, we keep *startPos *undefined for this unit.

Then during the backward search we track the number of terminal units we have seen so far in a variable called *termCount*, the maximum of all range maximum values seen so far in variable *rMax_max_*, and the corresponding minimum of the range minimum values in *rMin_min_*. We also track *minStartPos*, i.e., the last position in the list where a virtual gene was detected. The value of *termCount *is updated whenever we reach a new terminal unit, *rMax_max _*is updated when a non-terminal unit is processed and the maximum of the range maxima in the unit is bigger than the current *rMax_max_*. The value of *rMin_min _*is updated both at non-terminal units and at terminal units. In the latter case, however, the new minimum is only calculated between the current *rMin_min _*and the gene represented by the terminal unit. As a terminal gene, the occurrences of the next bigger gene on the chromosome, and of the genes located in between, are not relevant at this point.

Whenever we encounter two consecutive non-terminal units in the backward search, we merge them into a single unit. To do that we establish the minimum of their range minima and the maximum of their range maxima. We also need to update the value of *startPos*. If *startPos *of the older unit exists, it becomes this value, unless *rMin *of the newer unit is smaller than it. In that case it becomes the value of *startPos *of the newer unit, or undefined if that value does not exist.

New start positions of virtual intervals can be found at non-terminal and terminal units. In non-terminal units *startPos *needs to be defined and may not exceed the current *rMin_min _*to become the new minimal start position. In terminal units the gene of the unit may not exceed the current *rMin_min_*. After the backward search for a gene *g *is completed, the smallest start position of a virtual gene ending at *g *is combined with *g *and added to the list of virtual intervals. Once all *g *have been processed, we retrieve *L' *from this list as described earlier.

Apart from the merging of non-terminal intervals each backward search processes only a constant number of units. The total number of merging events between two non-terminal units is bound by the number of genes in *P*. Each processing of a unit and the merging of two units takes constant time. Thus the total runtime of the algorithm is in linear time with respect to the size of the gene set *L*.

**Table 1:** 

(Consolidation algorithm)
**Input: **diploid genome *D*, ancient (2*k*)-ploid genome *P*, gene set *L*
**Output: **gene set *L'*, each element of *L' *is a virtual gene shared between *D *and *P*, the set of the virtual genes forms a partition of *D *.
1: **for each **chromosome c in the diploid genome **do**
2: *recentTerm *← *undefined*
3: **for each **gene *g *on chromosome *c ***do**
4: **for each **occurrence *occ *of gene *g *on the hexaploid genome **do**
5: create new terminal unit *newTerm*, set link to previous terminal unit
6: **if ***newTerm *is not the first terminal unit on its chromosome **then**
7: turn the older terminal unit into non-terminal unit *newNonTerm*
8: update links between terminal units
9: set *startPos*(*newNonTerm*), *rMin*(*newNonTerm*), *rMax*(*newNonTerm*)
10: **end if**
11: *recentTerm *← *newTerm*
12: **end for**
13: [*rMin_min_, rMax_max_*] ← [*g, g*]
14: *currUnit *← *newTerm*
15: *termCount *←0
16: *minStartPos *← *undef*
17: **while ***currUnit *exists **and ***termCount *≤ *k *and *rMax_max _*≤ *g ***do**
18: **if ***currUnit *is a terminal gene **then**
19: *termCount *← *termCount *+ 1
20: *rMin_min _*← *min*(*rMin_min_*, gene of *currUnit*)
21: **if ***rMin_min _*≥ gene of *currUnit ***then**
22: *minStartPos *← gene of *currUnit*
23: **end if**
24: **end if**
25: **if ***currUnit *is a non-terminal unit **then**
26: **if ***rMax*(*currUnit*) ≤ *g ***then**
27: **while ***prevUnit*(*currUnit*) is non-terminal **and ***rMax*(*prevUnit*(*currUnit*)) ≤ *g ***do**
28: merge *prevUnit*(*currUnit*) into *currUnit*
29: update link to previous unit
30: update *startPos*(*currUnit*), *rMax*(*currUnit*) and *rMin*(*currUnit*)
31: **end while**
32: **if ***startPos*(*currUnit*) exists **and ***rMin_min _*≥ *startPos*(*currUnit*) **then**
33: *minStartPos *←*startPos*(*currUnit*)
34: **end if**
35: **end if**
36: *rMin_min _*← *min*(*rMin_min_*, *currUnit.rMin*)
37: *rMax_max _*← *max*(*rMax_max_*, *currUnit.rMax*)
38: **end if**
39: *currUnit *← *prevUnit*(*currUnit*)
40: **end while**
41: add [*minStartPos*, *g*] to list of virtual intervals
42: **end for**
43: **end for**
44: remove redundant intervals from list of virtual intervals to obtain a partition of *D*

### Allowing multiple gene copies on a chromosome

The challenge when allowing multiple copies of a gene on a chromosome is the following: if we have an occurrence *occ *of a gene *g *on a chromosome on which the next bigger gene *g' *after *g *occurs more than once, the range minimum and range maximum of *occ *may not be uniquely defined. We observe that at most two occurrences of *g' *are relevant for finding virtual intervals, namely the closest to the left and to the right of *occ*. The other occurrences could only be added to the same interval as *occ *when one of the closer occurrences is also added which is not possible as at most one occurrence of a gene is allowed per interval. We change the algorithm such that we initially keep both sets of range minimum and range maximum for gene occurrences affected by this effect. Once the smaller of the range maxima becomes smaller than the current *g*, *occ *might need to be merged with another non-terminal interval. We do this but remember at the same time that the occurrence may also be part of the other interval. Once we reach a g which equals the bigger of the two range maximum values of *occ *we switch it to the other interval, which means in fact only that we now use the other set of range minimum and range maximum for *occ*. If the range minimum of the new set is bigger than the range minimum of the old set, we may find a start position of a virtual interval earlier.

Before we do this swapping we need to check if structural constraints became available that already define whether *occ *belongs to the interval of the left or right occurrence of *g'*. For example if an occurrence of another gene to the right of *occ*, is the successor gene of a gene that is in the interval left of *occ*, *occ *can only be in this interval. Before making use of any of the range information provided by *occ *we need to make sure it is up to date with respect to the available structural constraints. Note that such structural information may be available already when *occ *is processed for the first time. Then *occ *can be processed like any other occurrence with only one set of range minimum and range maximum values.

Another novelty when allowing multiple gene copies on one chromosome is that terminal genes need not necessarily be transformed into non-terminal units immediately after the next bigger gene on the chromosome is processed. Instead they can remain terminal until the limited availablability of intervals *I*_2_, ... *I*_*k*+1 _makes it necessary to merge intervals to continue the backward search.

### Placing additional virtual genes

The consolidation algorithm we have presented may return virtual genes with less than *k *occurrences on the polyploid genome. We can place the missing occurrences in *P' *to transform it into a "pure" 2*k*-ploid and at the same time try reduce the number of (signed) adjacencies that are not shared between *D' *and *P' *. Since the genes within an occurrence of a virtual gene can be rearranged, we need to go back to the fractionation region on *D' *and *P' *that defines the virtual gene to decide whether the adjacencies between two pairs of neighboring virtual genes are the same. Assume we have the fractionation regions (5, 6, 7, 8)(9)(10, 11, 12), (13, 14), (15, 16) on *D *which occur as (−5, 6, 7)(10, −12, 11)(15, 16) on *P*. The adjacency between 7 and 10 can be explained by fractionation. We can add gene 8 at the end of the first interval as it is not yet present and then add a virtual occurrence of (9) after this interval. In doing so we create three adjacencies that are shared between the polyploid and the diploid and delete one adjacency that is not. The same is not possible between 11 and 15, as the genes in virtual gene (10, 11, 12) are rearranged on *P*. We can still place (13, 14) between the last two virtual genes, but the net reduction of gene adjacencies is zero.

To complete *P' *to a pure polyploid we first add missing genes within the fractionation regions preferring locations where the net reduction of gene adjacencies is one, then zero. Genes that would increase the net number of gene adjacencies are not added by default but could be if a complete reconstruction is desired. Then we add occurrences of virtual genes to *P' *to further reduce the number of adjacencies. For that purpose we count at each adjacency in *P' *how many virtual genes need to be added to obtain a positive net reduction of adjacencies. Then we start placing virtual genes at adjacencies with the smallest number of missing virtual genes while keeping track of the number of yet unplaced occurrences of each virtual gene to prevent having more than *k *copies in total. Once no more placements with a positive net reduction of adjacencies are possible, we continue with those of net gain zero. Optionally the remaining virtual genes that increase the number of adjacencies can be placed. This is a greedy approach that may not find an optimal solution in terms of reducing the number of adjacencies.

## Results and discussion

**Figure 2:**
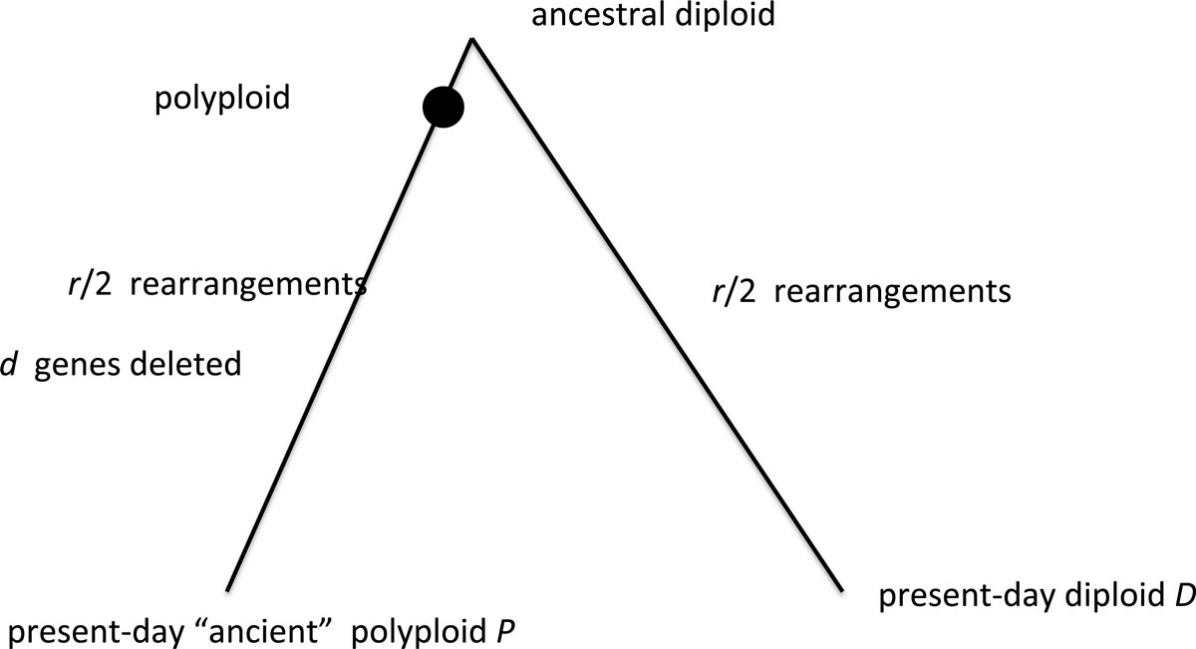
**Simulation Schema**. Schema for simulation of divergence between an ancient polyploid and a sister diploid.

To study the effect of fractionation on rearrangement distances in hexaploid genomes for different rates of gene deletion, we conducted a series of experiments on simulated data. Simulated genomes were generated based on the schema shown in Figure 2. An ancestral diploid with 9000 genes was generated and randomly distributed over 7 chromosomes, to simulate the ancestor of the core eudicots. The genome was triplicated in one lineage to generate the ancestral hexaploid, modeled after the grapevine. Genome evolution was simulated by random chromosomal inversions and reciprocal translocation in the proportions 20:1. Double deletion and single deletion of genes were applied in the proportion 5.5:1.

The simulations were repeated 10 times for each combination of *r *= 200, 600, 1000, 1400 rearrangements and *d *= 0, 1200, 3600, 5000, 6400, 7800 deletions, and the average over the 10 runs was plotted.

**Figure 3:**
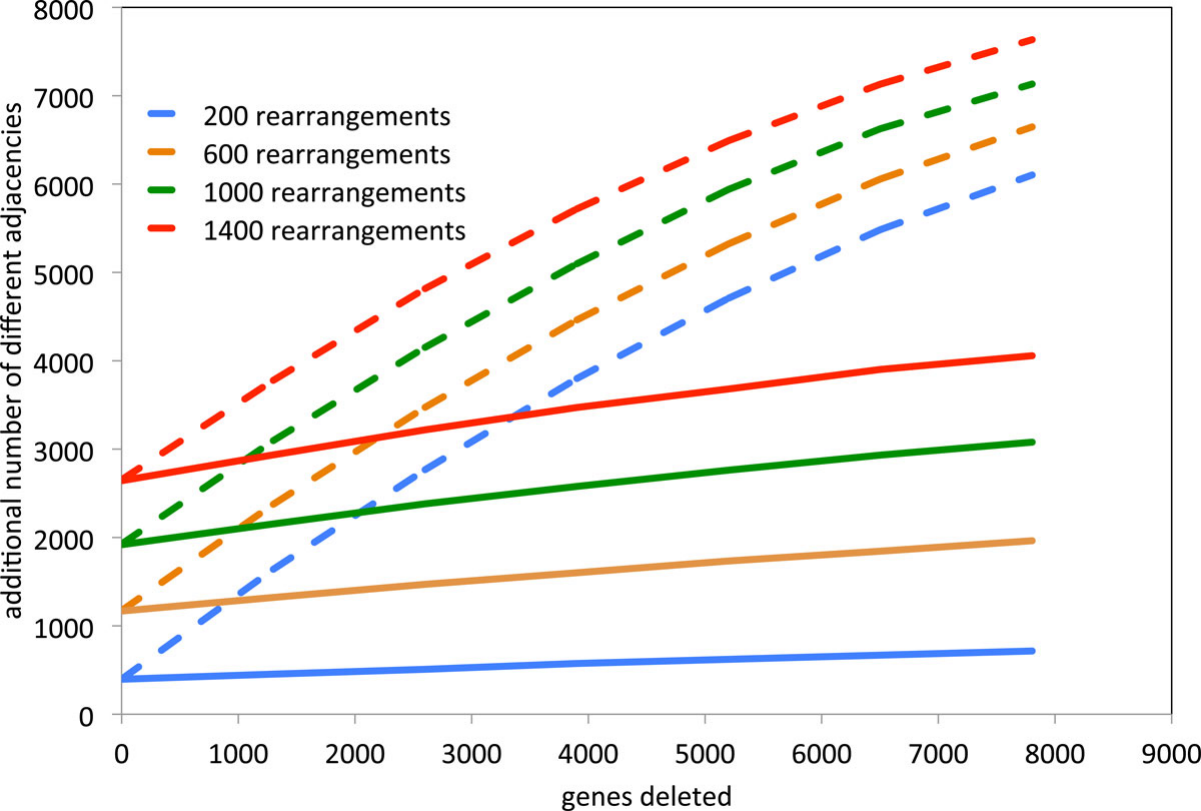
**Change in apparent rearrangement after application of the consolidation algorithm**. Change in apparent rearrangement in an ancient hexaploid compared to a diploid sister genome, as a function of actual rearrangements and number of deleted genes before (dashed lines) and after (solid lines) application of the consolidation algorithm.

The dashed lines in Figure 3 represent the apparent amount of rearrangement in the simulated genomes as a function of actual amount of rearrangement and the number of deleted gene copies. After applying the consolidation algorithm (solid lines), the apparent amount of rearrangement drops strongly and becomes almost independent on the number of deleted genes. These findings suggest that fractionation has the potential to cause a strong bias in rearrangement studies of ancient polyploids and, most important, that the consolidation is able to minimize this distortion. We have recently shown the same effect on simulated tetraploid data using the stricter definition of fractionation intervals where no duplicate genes are allowed in the fractionation region [8].

**Figure 4:**
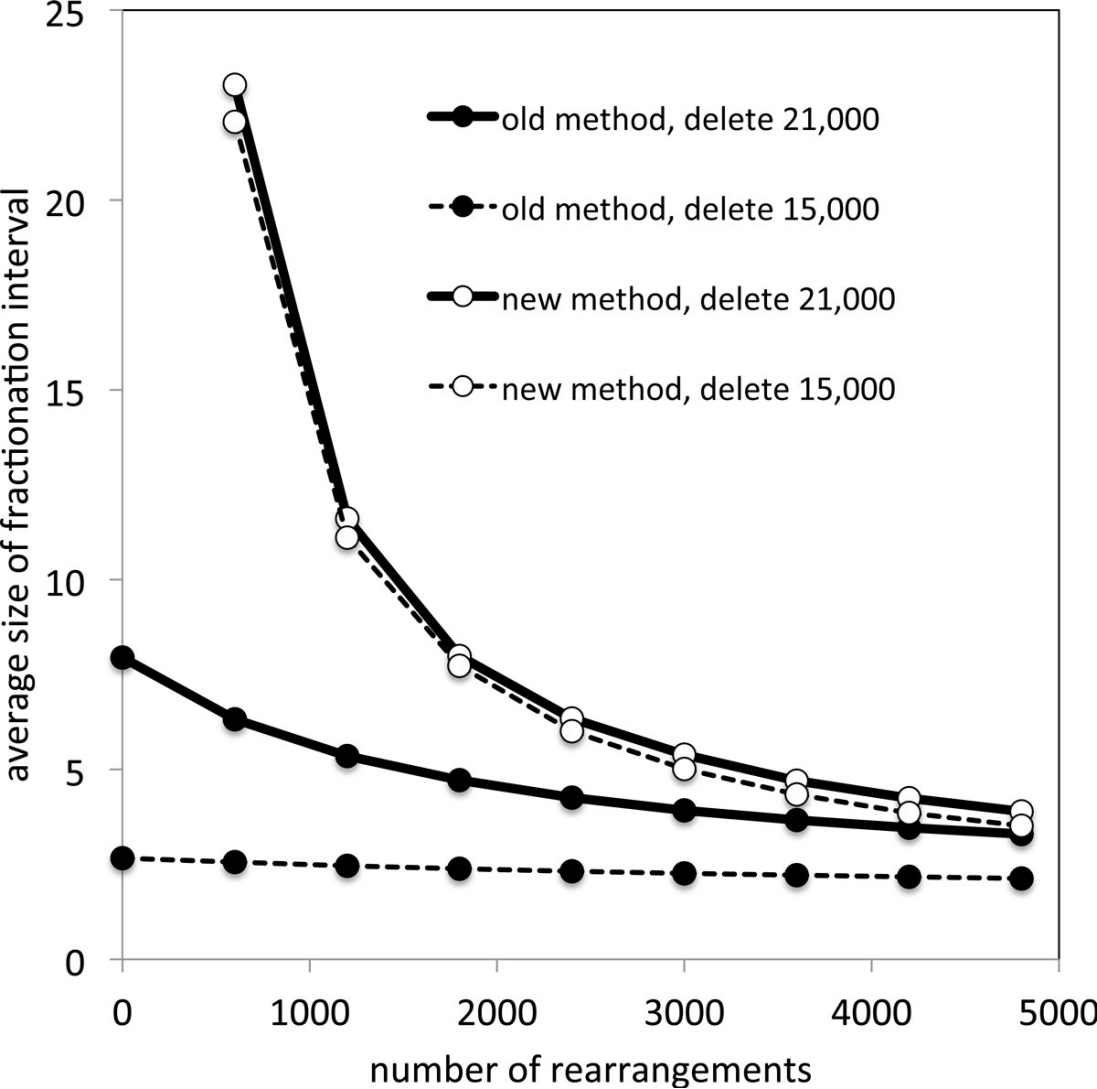
**Size of fractionation regions as a function of the number of rearrangements**. Size of fractionation regions as a function of the number of rearrangements for both definitions of fractionation regions on simulated tetraploid data

In a second experiment we studied how the choice of the definition of fractionation regions affects their average size. For this study we generated tetraploid genomes of length 24,000 with the above method and deleted 15,000, respectively 21,000 of the gene copies in the tetraploid lineage and using between 600 and 4800 rearrangements. Results of these experiments are plotted in Figure 4.

These results are the clearest consequences of the differences between the two definitions. In the old method, where fractionation regions contained only single-copy genes, these regions were short, and their length depended on the amount of deletion, but not very much on the amount of rearrangement. In the new definition, where fractionation regions can span both single- and double-copy genes, the regions are much longer, there is virtually no effect of the amount of deletion, but a strong dependence on the number of rearrangements. This reflects the exclusive role of rearrangement breakpoints in the new definition, whereas the old definition also depended on adjacencies between single-copy and multiple-copy genes.

Though analyzing the simulations has been instructive, to investigate fractionation in the grapevine, as an ancient hexaploid, would eventually require access to a diploid genome from an outgroup of the core eudicots.

**Figure 5:**
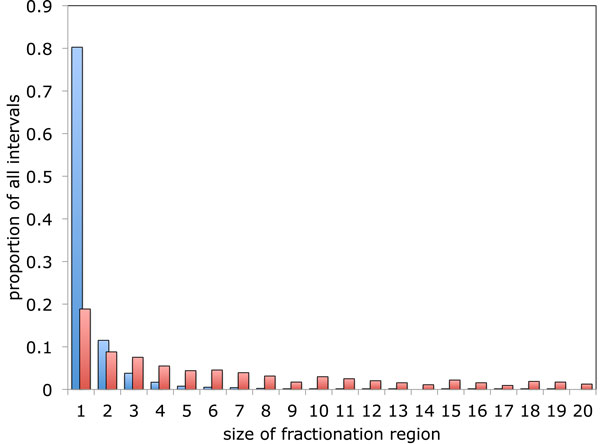
**Size distribution of Populus-Vitis fractionation regions**. Size distribution of *Populus-Vitis *fractionation regions. Blue bars: distribution generated with the original definition where fractionation intervals may only contain single copy genes. Red bars represent the generalized definition that allows multiple copies of a gene if they occur in different intervals.

We have, nonetheless, been able to study the effect of the choice of the fractionation interval definition on real genomes. The bar plot in Figure 5 shows the relative frequency of different fractionation region sizes in the comparison of the ancient tetraploid poplar (*Populus trichocarpa*) to the diploid sister genome grapevine (*Vitis vinifera*). Note that the polyploidy studied here is relatively recent compared to the core eudicot hexaploidy shared by both genomes, so that the older gene pairs and triples are easily filtered out using gene sequence comparison measures.

This graph gives a more detailed picture of the consequences of weakening the definition of the fractionation interval, when compared to the previous study [8]. The distribution of sizes with the new definition not only has a higher average values, but is much flatter than with the old one.

## Conclusions

The main contribution of this work is the algorithm that reduces computing time from quadratic to linear in *n*, the number of genes in the genome, and that applies to polyploids of any order, not just tetraploids. Thus we were able to analyze fractionation triggered by the all-important hexaploidization event at the base of the core eudicots.

The previous definition of a fractionation region required it to contain only single-copy genes. We have proposed to weaken this restriction, allowing multi-copy genes to appear in corresponding fractionation intervals in the ancient polyploid, as long as each undeleted copy of a gene is contained in a different such interval. This weakening of the condition is not a necessary aspect of the new algorithm, and both notions of fractionation interval have their advantages. The stronger restriction may be of more interest to biologists interested specifically in single-copy regions of paleopolyploid genomes. The weaker restriction is more closely tied to the analysis of genome rearrangement since the boundaries of intervals are now necessarily rearrangement breakpoints (or chromosome ends) and not necessarily adjacencies with multi-copy genes. Moreover, under the new definition, the sizes of fractionation intervals are insensitive to the number of deletions, a property which may be useful for some research goals but detrimental for others.. The fractionation interval under both conceptions resembles "conserved intervals" in that all rearrangements have either operated within such an interval or have left these intervals intact, either because the intervals are outside the scope of the rearrangement or the interval is affected as a whole, without any effect internally. The difference is that in the new, weaker, definition, the entire paleopolyploid genome is decomposed into intervals, not just the single-copy regions.

A temporary impediment to applying this work is the current unavailability of published flowering plant genomes that have escaped polyploidization since the inception of this clade, but this difficulty should be resolved in the near future with the publication of the *Amborella trichopoda *genome [13].

## Authors' contributions

CZ, JK and DS worked on a linear time algorithm for the original consolidation problem. KJ generalized the problem definition and developed the algorithm presented in this paper. KJ, CZ and DS designed and conducted the experimental study. KJ and DS drafted the manuscript. All authors read and approved the final manuscript.

## Competing interests

The authors declare that they have no competing interests.

## Endnotes

*^a^*The terminology for polyploids (di-, tetra-, hexa-, ...) reflects the number of chromosomes in the non-meiotic plant cell nucleus, with two copies, maternal and paternal, of each chromosome. Working on the evolutionary time scale, differences between these two are negligible, so it suffices to study the haploid (*k *= 1, 2, 3, …, respectively).
